# Induction of apoptosis by pinostrobin in human cervical cancer cells: Possible mechanism of action

**DOI:** 10.1371/journal.pone.0191523

**Published:** 2018-02-08

**Authors:** Alka Jaudan, Sapna Sharma, Sri Nurestri Abd Malek, Aparna Dixit

**Affiliations:** 1 Gene Regulation Laboratory, School of Biotechnology, Jawaharlal Nehru University, New Delhi, India; 2 Faculty of Science, University of Malaya, Kuala Lumpur, Malaysia; Institute of Biochemistry and Biotechnology, TAIWAN

## Abstract

Pinostrobin (P_N_) is a naturally occurring dietary bioflavonoid, found in various medicinal herbs/plants. Though anti-cancer potential of many such similar constituents has been demonstrated, critical biochemical targets and exact mechanism for their apoptosis-inducing actions have not been fully elucidated. The present study was aimed to investigate if P_N_ induced apoptosis in cervical cancer cells (HeLa) of human origin. It is demonstrated that P_N_ at increasing dose effectivity reduced the cell viability as well as GSH and NO_2_^-^ levels. Condensed nuclei with fragmented chromatin and changes in mitochondrial matrix morphology clearly indicated the role of mitochondria in P_N_ induced apoptosis. A marked reduction in mitochondrial membrane potential and increased ROS production after P_N_ treatment showed involvement of free radicals, which in turn further augment ROS levels. P_N_ treatment resulted in DNA damage, which could have been triggered by an increase in ROS levels. Decrease in apoptotic cells in the presence of caspase 3 inhibitor in P_N_-treated cells suggested that P_N_ induced apoptosis via caspase dependent pathways. Additionally, a significant increase in the expression of proteins of extrinsic (TRAIL R1/DR4, TRAIL R2/DR5, TNF RI/TNFRSF1A, FADD, Fas/TNFRSF6) and intrinsic pathway (Bad, Bax, HTRA2/Omi, SMAC/Diablo, cytochrome C, Pro-Caspase-3, Cleaved Caspase-3) was observed in the cells exposed to P_N_. Taken together, these observations suggest that P_N_ efficiently induces apoptosis through ROS mediated extrinsic and intrinsic dependent signaling pathways, as well as ROS mediated mitochondrial damage in HeLa cells.

## Introduction

According to the World Health Organization (WHO), cervical cancer is globally the second most prevalent cancer in women with an estimated 44, 5000 new cases in every year [1]. Cervical cancer is a consequence of a long-term infection with human papillomavirus (HPV), and the majority of cases (>84%) occur in low- and middle-income countries. Of 270,000 deaths resulting from cervical cancer worldwide, approximately 85% of these occur in developing countries [1]. HPV infection proceeds by integration of its genome into that of host’s, leading to dysregulation of cellular processes. These include increased DNA synthesis, cell proliferation, and altered cellular response to growth and differentiation factors, which eventually lead to the development of cervical cancer and reoccurrence [2]. However, majority of HPV infections do not cause symptoms/disease and oncogenic HPV infection alone is not responsible for tumor development. Therefore, other factors are likely to be involved in the progression of the infected cells to the full neoplastic phenotype. Significant changes in levels of oxidative and nitrosative stress indicators have been observed in cervical cancer patients [3]. Dysplastic cervical tissues lesions (CIN2/3) and invasive squamous cervical carcinoma tissues showed significantly higher expression of protein carbonyls [4]. Imbalance in the redox status of the cell causes detrimental oxidative stress leading to cell death. ROS can induce genotoxic damage, including single- and double-strand breaks, DNA-protein cross-links, basic sites and modified bases [5]. Several *in-vitro* studies have demonstrated that apoptosis was accompanied by down-regulation of Bcl-2, up-regulation of Bax, release of cytochrome c from mitochondria, activation of caspase-9 and caspase-3, and subsequently inhibited cell proliferation through G0/G1 cell cycle arrest, and induced apoptosis via the mitochondrial apoptotic pathway in human cervical cancer cells [6–8]. Recent evidences suggested that apoptotic pathways coincide at the mitochondria, where signaling is initiated through a series of molecular events which begin with the release of death factors [7, 9], this triggers either caspase-dependent or independent apoptosis. Mitochondrial apoptotic proteins like cytochrome *c* (Cyt *c*), causes caspase-dependent cell death and activates caspase-9 activation by binding and activating apoptotic protease activating factor-1 (ApoAF-1). This event potentiates caspase activation by binding inhibitor of apoptosis proteins (IAP) and blocking their caspase-inhibitory activity [9]. Apoptosis-inducing factor (AIF) and endonuclease G (EndoG), along with other important mitochondrial proapoptotic proteins, are reported to translocate to the nucleus and cause oligonucleosomal DNA fragmentation during mitochondria mediated caspase-independent cell death [10]. Release of these mitochondrial death effectors are tightly regulated by the Bcl-2 family proteins during membrane permeability transition (MPT). It is clearly a redox sensitive phenomenon in which membrane protein thiols get oxidized and cross-linked; leading to increased mitochondrial membrane permeability [9, 10]. This phenomenon is also sensitive to glutathione depletion in a synchronized manner with oxidative stress and calcium overload.

Despite advances in the treatment of cervical cancers, protocols for recurrent or persistent cancers and alternative treatment options with low toxicity are still on demand. Because better understanding of the molecular mechanisms of drug action has shed light on the treatment of cervical cancer, novel agents that target specific intracellular pathways related to the distinctive properties of cancer cells continue to be developed. Cancer therapy has changed substantially during the past decades because many new types of therapy including monoclonal antibodies and targeted anticancer drugs have been introduced. Although three vaccines against HPV are available against type 16 and 18, however vaccination among immunocompromised patients is still challenging both with respect to efficacy and safety. The background and characteristics of the immunosuppressed states differ between different patient groups. Vaccination against the HPV gave weaker responses in children with ongoing chemotherapy [11].The timing of vaccination in cancer patients is important for the response, considering varied effects of vaccination, chemotherapy continues to be the method for treating HPV-infected patients. However, acquired chemo-resistivity of cancer cells that leads into the decreased therapeutic efficacy is the major impediment to conventional cancer chemotherapy.

Use of natural constituents as promising chemo-preventive agent against cancer has been extensively studied in the last two decades [12]. Studies have suggested that the use of anti-cancer phytochemicals with different mechanisms or modes of action may be more effective in treating the diseases and limiting the side-effects [12, 13]. Flavonoids are a group of polyphenolic compounds of low molecular weight that are present in food and exhibit a common benzo-γ-pyrone structure. They are further sub-categorized into various subclasses including flavones, flavonols, flavanones, isoflavanones, anthocyanidins, and catechins [13]. A number of flavonoids have been reported to promote cell death by perturbing the cell cycle progression [14]. Pinostrobin (P_N_) (5-hydroxy-7-methoxy flavanone) and their corresponding glycosides belong to the flavanone family extracted from several medicinal plants [15] and are also present in commercial nutraceuticals [16]. P_N_ has been found to exhibit anti-*Helicobacter pylori*, trypanocidal activity and anti-herpes simplex virus-1 activity [17–19]. P_N_ has been reported to inhibit DNA topoisomerase I activity *in vitro* [20].

Few reports have been put forth regarding anti-cancer and anti-proliferative activity of P_N,_ little is known about its mechanism of action. In the present study, we have made an attempt to investigate the ability of P_N_ to modulate the endogenous antioxidative systems in HeLa cells as a possible underlying mechanism of action. Further, expression analysis of different proteins involved in regulation of apoptotic signaling pathway was also carried out to gain an insight into the pathway by which P_N_ induced apoptosis.

## Materials and methods

### Reagents

All reagents used in the present study were of molecular biology grade and were procured from Sigma Aldrich Chemical Co., USA. Pinostrobin (Cat no. 38790) was purchased from Sigma-Aldrich Chemical Co., (USA). Various solvents of ultrapure/cell culture grade were purchased from Merck, India. Triple solvent containing dimethyl sulfoxide: dimethyl formamide: acetonitrile in equal ratio was used to prepare P_N_ stock solution [20].

### Cell cultures

HeLa (human adeno cervical carcinoma cells), Ca Ski (human epidermoid cervical carcinoma cells), SiHa (Grade II, squamous cervical carcinoma cell) and HEK293 (Human embryonic kidney cells) cell lines were procured from culture collection at National Centre for Cell Science (NCCS, Pune) India. HeLa, SiHa and HEK293 cell lines were cultured and maintained in DMEM supplemented with 10% FBS whereas Ca Ski cells were maintained in RPMI medium supplemented with 10% FBS. The cultures were maintained in the presence of penicillin (100 U/ml) and streptomycin (100 μg/ml) in a humidified atmosphere of 95% air and in 5% CO_2_ incubator (HERA Cell-150-Thermo Fisher Scientific, USA).

### Assessment of cell viability

The cytotoxic effect of P_N_ was assessed by performing MTT reduction assay [21]. The cells were seeded at a density of 5x10^3^ cells/well in 96 well plates (Greiner, Germany) and incubated for 24 h in CO_2_ incubator. The cells were then treated with various concentrations of P_N_ (10, 30, 50, 100, 200 μM, in triplicate). Cells treated with triple solvent (vehicle) and Doxorubicin (D_X_, 10 μM) was included as negative and positive controls, respectively. Cell viability was monitored at 24, 48, 72 and 96 h post-treatment using MTT assay essentially as described [21]. Absorbance was measured at 570 nm using a microplate ELISA reader (Tecan, USA). The data was expressed as viability (%) as compared to vehicle treated cells.

Since HeLa cells showed minimum CT_50_ concentration (i.e. 50 μM) in comparison to other two test cell lines (CT_50_ of CaSki and SiHa, 75 μM and 100 μM, respectively), therefore further studies were performed using HeLa cells only.

### Morphological Assessment

All imaging analysis was performed at the Advanced Instruments Research Facility (AIRF) of the Jawaharlal Nehru University, New Delhi, India.

#### Wright-Giemsa staining

Morphological alteration in nucleus and cells was investigated by Wright-Giemsa staining [22]. HeLa cells (5×10^4^ cells/2mL/well) were grown on 24 mm cover slip in 6-well plates treated with P_N_ (pre-determined CT_50_) and D_X_ (10 μM) for 48 h. After treatment, cells were washed with PBS (pH 7.4) and stained with Wright-Giemsa for 5 min at RT and washed once with PBS. Eventually, morphological changes were examined under light microscope at 40× magnification (Nikon TMS).

#### Acridine orange/ethidium bromide staining (AO/EB staining)

Acridine orange/ethidium bromide staining was performed according to the method of Ariffin *et al*., [22]. Morphology of HeLa cells treated with P_N_ (50 μM, 2×50 μM) for 48 h was visualized and imaged under confocal Laser-Scanning microscope (Olympus Fluroview FV1000) at 40× magnifications using 1.4NA oil objective.

#### Transmission Electron Microscopy (TEM)

TEM study was performed by following Altinoz’s method [23]. HeLa cells, treated with P_N_ (50 μM, 2× 50 μM) and D_X_ (10 μM) and incubated for 48 h were examined at direct 15000× magnifications under Transmission Electron Microscope (TEM, JEOL 2100F).

### Live cell imaging microscopic studies

Live cell imaging microscopic study was performed to visualize the internalization and retention of P_N_ within the cells [24]. HeLa cells (5×10^4^ cells/well) were cultured on glass culture dish (33 mm) and treated with P_N_ (100 μM). Culture dish was kept in an enclosed chamber of live cell confocal microscope (Nikon, A1R eclipse-Ti) for providing an adequate cell culture conditions (37°C temperature with 5% CO_2_) with all other parameters with default setting. Live P_N_ internalization, retention into nucleus from cytoplasm and apoptosis induction were visualized by NIS-Elements imaging software at 1h and 30 h post P_N_-treatment. In addition, we also determined the effect of P_N_ on deformation of nucleus structure and mitochondrial membrane potential using Hoechst 33342 stain (100 μg/ml) and JC-1 probes (1 μg/ml) according to the manufacturer’s instructions [25]. Morphology of stained cells was visualized and imaged at 40× magnification under live cell confocal microscopy.

### Estimation of intracellular nitrite (NO_2_^-^) and reduced glutathione (GSH) levels

Enzymatic and non-enzymatic defense system was majorly involved in the oxidative stress implications. Nitric oxide (NO˙) acts as signaling molecule either react with oxygen or other free radical to form RNS (Reactive nitrogen species) which causes multiple biological effects [26]. GSH plays major role in protection against oxidative stress and depletion of GSH levels could be due to higher production of ROS [5]. Thus, depletion of GSH could enhance the susceptibility of oxidative stress mediated by ROS, ultimately facilitating cell death [27]. Therefore, changes in the cellular concentration of ubiquitously present signaling molecules such as nitrite and GSH were estimated by Griess and DTNB (5–5'-Dithiobis 2-nitrobenzoic acid) reagents, respectively [28, 29]. For this, cells were seeded at a density of 5×10^4^ cells/ well in 24 well culture plate and incubated with P_N_ (50 μM, 2×50 μM) and D_X_ (10 μM) for 48 h. For NO_2_^**-**^ measurements, we collect culture supernatant (100 μl) and mixed with equal volume of Griess reagent simultaneously for 10 min at RT and absorbance was measured at 540 nm using microplate ELISA reader (Tecan, USA).

GSH levels can be measured using 5–5'-Dithiobis 2-nitrobenzoic acid (DTNB, Ellman’s reagent). Reduced glutathione (GSH) reacts with DTNB and produces TMB (tetramethylbenzidine) chromophore at 412 nm. Cells were trypsinized and lysed using RIPA buffer (100 μl) followed by centrifugation at 8000 rpm for 10 min at 4°C. Clear supernatant, (100 μl) thus obtained was mixed with phosphate buffer (0.2 M, pH 8) containing DTNB (0.6 mM) [29]. An aliquot from the mix was used for measurement of absorbance at 412 nm in a microplate ELISA reader. Reduced GSH (187.5 μg/ml) was used to obtained standard curve and GSH was expressed in μg/mg protein in all experimental groups.

### Flow cytometric analysis

HeLa cells (5x10^5^/ml/well) were seeded in 24 well plates and incubated for next 24 h. Further cultured cells were incubated with P_N_ (50 μM, 2×50 μM) for additional 48 h. Cells treated with D_X_ (10 μM) were included as positive control. Subsequently, the treated cells were subjected to staining with different fluorescent dyes as detailed below:

#### Measurement of intracellular Reactive Oxygen Species (ROS) production

Fluorescent probe DCFH-DA (2′, 7-dichlorofluoresceindiacetate) was used to measure ROS generation in HeLa cells after treatment with P_N_, by the method of Zhao *et al*., 2015 [30]. Upon entering the cell, the diacetate bond of the fluoroprobe is cleaved by intracellular esterase leaving DCFH which is oxidized to DCF (dichlorofluorescein) by the oxidants and its fluorescence is taken as an indicator of ROS production in the cell. Mean fluorescence intensity (MFI) was measured by FACS Calibur^TM^ (Becton Dickinson, SanJose, CA, USA) and analyzed on FCS Express.v5 Flow Cytometry data analysis software.

#### Assessment of mitochondrial membrane potential *(ΔΨm*)

Flouroprobe JC-1 (5, 5′, 6, 6′-tetrachloro-1, 1′, 3, 3′tetraethylbenzimidazol-carbocyanine iodide) has been extensively used to study the loss of the mitochondrial membrane potential which occurs during apoptosis. P_N_-treated cells were washed with 1×PBS twice and incubated with JC-1 dye (1 μg/ml) for 30 min at 37°C in dark. After washing the cells with 1×PBS, *ΔΨm* was assessed by comparing two fluorescence, i.e. red (Ex/Em-580/590 nm)/ green (Ex/Em-510/527 nm) using flow cytometer (FACS Calibur^TM^) and FCS Express.v5 Flow Cytometry data analysis software [25].

### Determination of apoptosis

#### Propidium iodide (PI) staining

DNA content from the HeLa cells was quantified using stochiometric dye, propidium iodide (PI) after P_N-_ treatment as described earlier by Yong and Abd Malek with minor modifications [25]. The cells were fixed with 2% paraformaldehyde (PFA) and suspended in 1 ml ice cold 1×PBS. PI master mix [500μl; prepared in 1×PBS buffer containing DNase-free RNase (10 μg/ml), PI (100 μg/ml) and 0.1% Triton×100] was then added to the cells and allowed to incubated in dark for 30 min at 37°C followed by twice 1×PBS wash. Finally fluorescence was measured by FACS Calibur^TM^ and analyzed on FCS Express.v5 Flow Cytometry data analysis software.

#### AnnexinV-FITC staining

HeLa cells treated with P_N_ and D_X_ were harvested by centrifugation at 300×g for 5 min and suspended in 1×annexin binding buffer (10×buffer composition: 0.1 M hepes/NaOH (pH 7.4), 1.4 M NaCl, 25mM CaCl_2_). Subsequently, annexinV-FITC reagent (5 μl) was added to the cell suspension and incubated for 20 min at 37°C in dark [25]. Final fluorescence of annexinV-FITC was acquired in 10,000 events on FL1 channel by FACS Calibur^TM^ and analyzed by FCS Express.v5 Flow Cytometry data analysis software.

#### Transferase Biotin-dUTP Nick End Labeling (TUNEL) assay

DNA fragmentation in apoptotic cells was detected by HT Titer TACSTM Assay Kit (Trevigen, Maryland, USA). Cells treated with P_N_ (50 μM, 2×50 μM) in the presence or absence of caspases 3 inhibitor (50 μM; Z-DEVD-FMK-Caspase-3 Inhibitor) for 48 h, harvested as described earlier, washed once with PBS and fixed in 3.7% buffered formaldehyde for 10 min. Cells were then washed twice with PBS, fixed with 100% methanol for 20 min. DNA labelling using the HT Titer TACSTM kit was carried out according to the manufacturer instructions. Fluorescence was measured at 450 nm using microplate ELISA reader to determine cell apoptotic unit.

### Cell cycle analysis

HeLa cells were cultured overnight on sterile culture plates and treated with P_N_ (50 μM, 2×50 μM), D_X_ (10 μM) and vehicle for 48 h in a 37°C and 5% CO_2_ incubator. The cells were harvested by centrifugation, washed with 1×PBS, and fixed in 4% PFA for 30 min at RT. The PFA-fixed cells were then pelleted, washed with 1×PBS, and suspended in a staining solution containing PI (50 μg/ml), RNase (100 μg/ml), sodium citrate (0.1%), and triton×100 (0.1%) for 30 min [25]. Further, the cells cycle analysis was carried out by flow cytometry (FACS Calibur^TM^).

### Apoptosis protein profiling study

Cell lysate prepared from P_N_-treated cells (50 μM, 2×50 μM), at 48 h post-treatment was analyzed for relative levels of apoptosis-related proteins using Proteome Profiler antibody array (R&D Systems, USA) as per the manufacturer’s instructions. Cell lysates from the control and treated cells were prepared using RIPA buffer in presence of 1% cocktail protease inhibitor. BCA Protein assay kit (Pierce, USA) was used to determine protein concentration. Levels of different apoptosis related proteins in the cell lysates were determined using the ready-to-use pre-coated array membranes were blocked for 1×array buffer 1 as described in manual instruction. Blocking buffer was then removed, and the cell lysate (1.25 ml of cell lysate added to 1.5 ml of lysis buffer 17, provided with the kit) was incubated overnight at 4°C on a rocking platform shaker. After washing with 1×wash buffer (20 ml) for 10 min, membranes were incubated for 1h at RT with 15 μl of reconstituted detection antibody cocktail (1.5 ml in array buffer 2/3) with shaking. The membrane was again washed with 1×wash buffer, and further incubated with diluted streptavidin-HRP for 30 min on shaking. The excess buffer was removed and immune reactive spots were detected by the addition of 1 ml of Chemi Reagent mix (provided in the kit) for 1 min. The images were captured by a ChemiDoc, Biospectrum-500 (UVP, USA), and densitometric analysis of the immunoreactive spots was performed using Vision Works® LS analysis software version 6.8 (UVP Laboratory Products, USA).

### Statistical analysis

Statistical analyses were carried out using GraphPad software (Chicago, USA). All experimental results are expressed as mean±SD from at least three independent experiments, performed in triplicates.

## Results

### Inhibition of cell proliferation by P_N_

**Fig 1:**
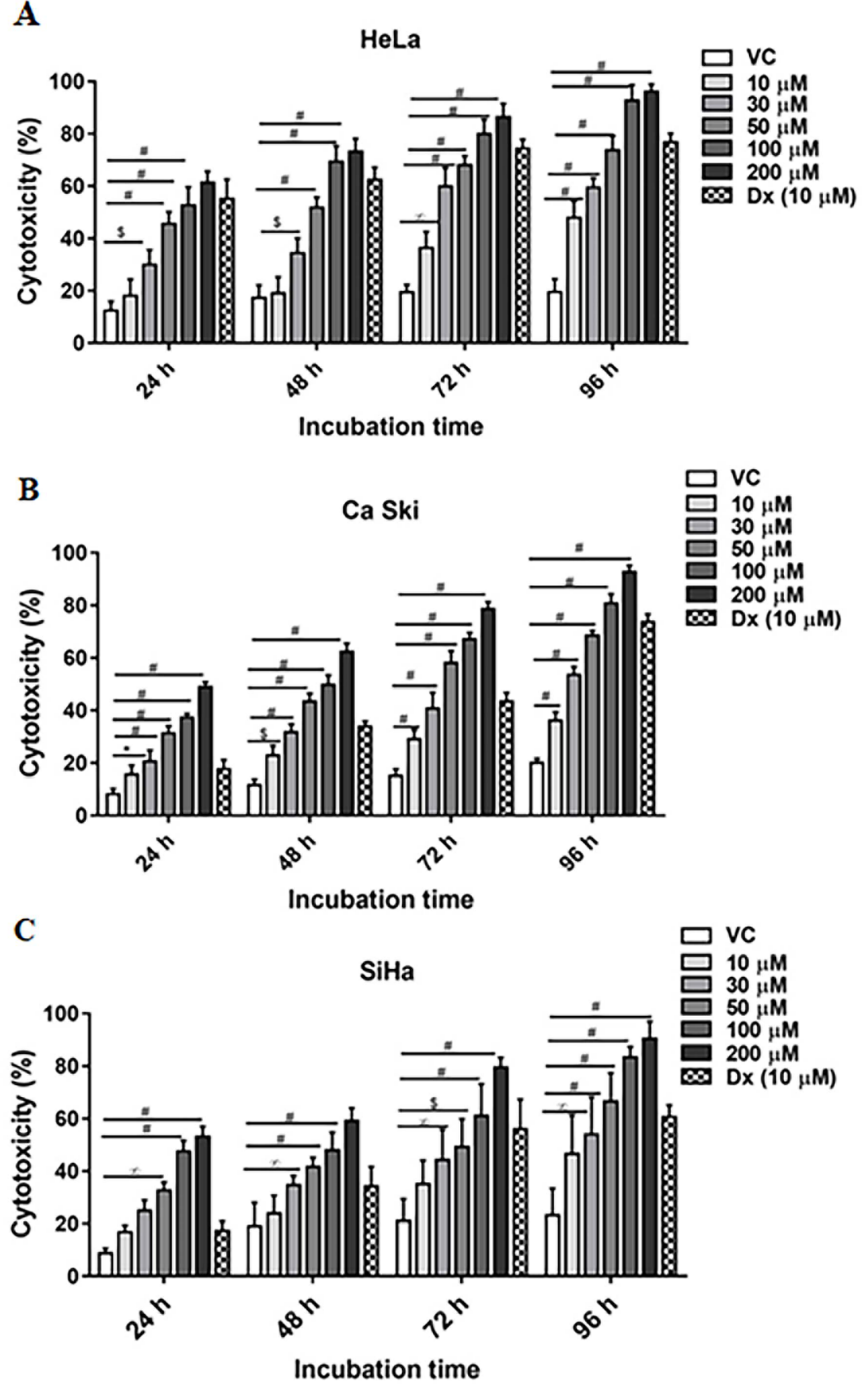
Impact of P_N_ on cell viability and morphology. Cytotoxicity % (CT) was assessed in cervical cell lines (A) HeLa, (B) Ca Ski, (C) SiHa cells on different concentrations of P_N_ treatments as determined by MTT reduction assay at different incubation period. The bar graphs represent the percentage of cytotoxicity of P_N_ in the cells. Cytotoxicity is shown as mean ± SD derived from at least three separate experiments in triplicate wells. Ordinary two-way ANOVA (multiple comparisons) was performed to calculate the statistical difference (*p≤0*.*05*) among all treated groups as compared to vehicle treated group. * represents *p*≤0.05, ∞ *p*≤0.01, $ *p*≤0.001 and # *p*≤0.0001.

In order to evaluate the cytotoxicity of P_N_ in different cervical cancer cells, effect of P_N_ on the cell viability was evaluated using MTT reduction assay. Viable cells population was significantly reduced after exposure of different doses of P_N_ (Fig 1). Of the three different cervical cancer cell lines, P_N_ showed highest toxicity for HeLa cells followed by Ca Ski and SiHa (Fig 1). The CT_50_ value of P_N_ for HeLa was determined to be 50μM (*p≤0*.*001*), whereas, in Ca Ski and SiHa cell lines 50% inhibition in cell viability is observed at 75 and 100 μM (*p≤0*.*001*) at 48 h, respectively. Time dependence of the cytotoxic effect of P_N_ was also assessed by incubating the cells for different time periods (24, 48, 72, 96 h). Fig 1 clearly illustrated that higher cytotoxicity was observed with longer incubation period in all the three cell lines. For confirmation of cytopathic potential of P_N_ against cancer cells, D_X_ a well-known anticancer agent included as a positive control, also showed cytotoxicity up to 62.4±4.7% (*p≤0*.*001*), 33.86±2.0% (*p≤0*.*001*) and 34.23±7.4% (*p≤0*.*05*) in HeLa, Ca Ski, SiHa respectively after 48 hours of incubation. Notably, vehicle control did not show any significant toxicity for cells. In contrast, we also determined the P_N_ cytotoxicity on HEK 293, non-cancerous cells and found negligible toxicity at higher concentrations (S1 Fig).

### P_N_ exhilarated apoptotic cell death

**Fig 2:**
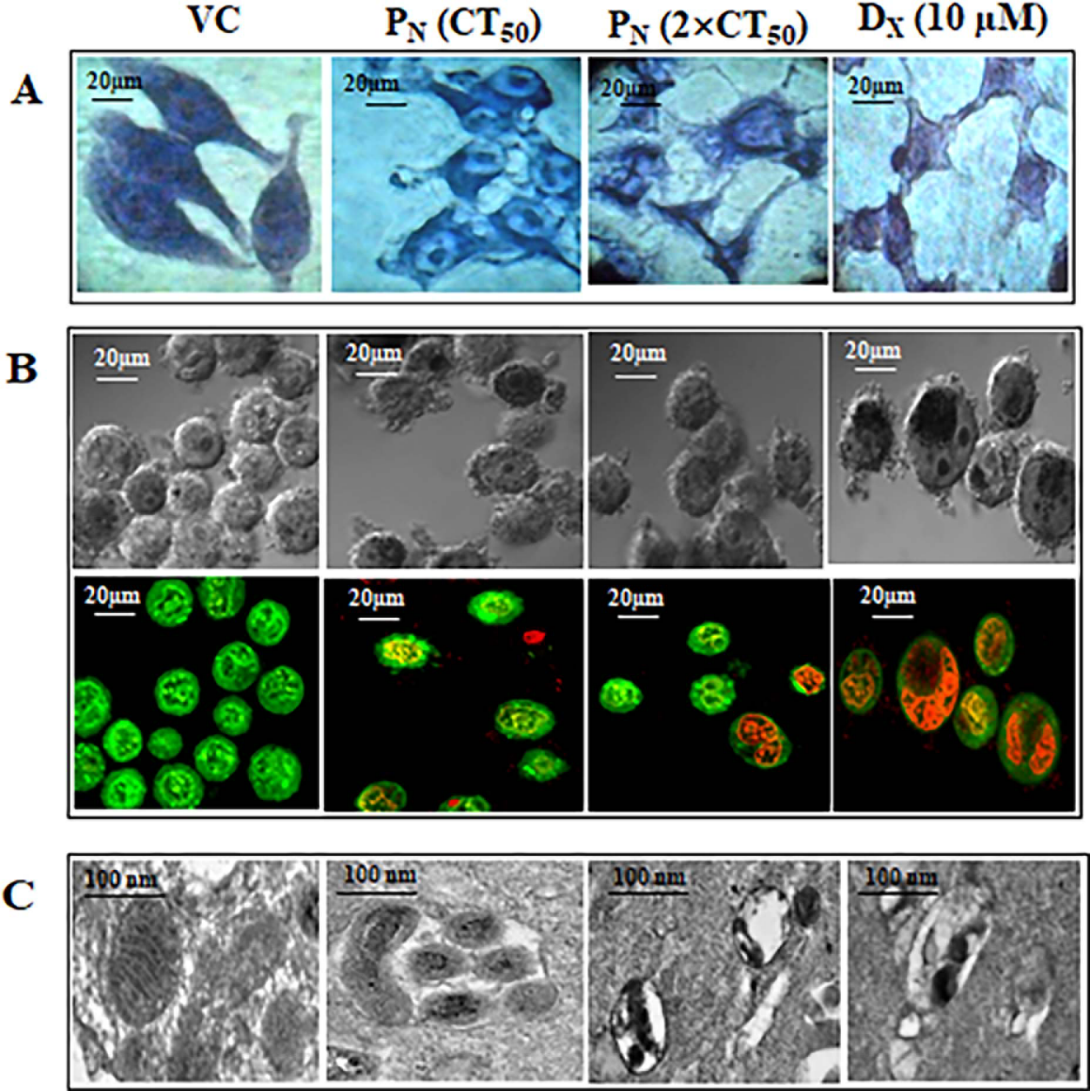
Representative photomicrographs of morphology of HeLa cells stained with (A) Giemsa (B) Acridine orange with ethidium bromide, fluorescent microscopy showing intracellular entry of P_N_ at 48h and visualized at 40 × magnifications_._ (C) Electron micrograph of ultra-thin section of VC, P_N_ (CT_50_ and 2×CT_50_) and D_X_ treated HeLa cells are shown at 48h and visualized at 15000× magnifications. Mitochondria show an interconnected network structure with numerous regularly arranged cristae, i.e., intact/condensed structures in VC compared to P_N_ and D_X_ treated cells. P_N:_ Pinostrobin, D_X:_ Doxorubicin, VC: Vehicle control.

In order to confirm the cytopathic effect of P_N_ on HeLa cells and to establish if the cell death is due to apoptosis or necrosis, morphological examination of the P_N_-treated cells was conducted after Geimsa staining using light microscopy for detection of nuclear condensation as biomarker of DNA damage (Fig 2). Giemsa- stained P_N_-treated cells showed remarkable changes in morphology such as cell shrinkage, condensed nuclei, membrane blebbing and apoptotic bodies formation at 48h, whereas vehicle treated cells displayed normal morphology intact nuclear and cellular architecture, typical of HeLa cells (Fig 2A).

To further confirm P_N_-induced apoptosis, to assess the extent of deformation of nuclear structure, and to distinguish between early and late apoptotic cell populations, AO/EB staining of the P_N_-treated HeLa cells was carried out. P_N_ (50 μM & 100 μM) and D_X_ (10μM) treated HeLa cells exhibited condensed nuclei with fragmented chromatin stained from orange to red color fragmented chromatin indicating apoptosis (Fig 2B). Some of the treated cells and nuclei were found to be disintegrated and fragmented into small spherical fragments, suggesting formation of apoptosis bodies. AO stained cells (fluorescent green) showed early apoptotic cells with intact membrane. Treatment with 2×CT_50_ concentration of P_N,_ resulted in higher number of late apoptotic cells as evidenced by orange-red staining of cells by EB due to damaged membrane [31]. Such color distribution was not observed in vehicle treated cells which showed green nuclei with intact cell/nuclei structure (Fig 2B). From these observations, we conclude that P_N_ strongly affects the morphology of nucleus that is associated with apoptosis. Morphological changes of nucleus, mitochondria and cells structure were also examined under TEM microscopy (Fig 2C). Electron microscopic tomography of P_N_-treated HeLa cells showed remodeling of the inner mitochondrial membrane, vesiculation and swelling of the mitochondrial matrix identified by less cristae and expanded matrix space. Swelling of the mitochondria resulted in disintegration of the mitochondrial membrane. On the other hand, the vehicle treated cells showed normal matrix compartments with uniform crista junctions and no vesiculation in the mitochondria.

### P_N_ internalization leads to cells structural deformity

**Fig 3:**
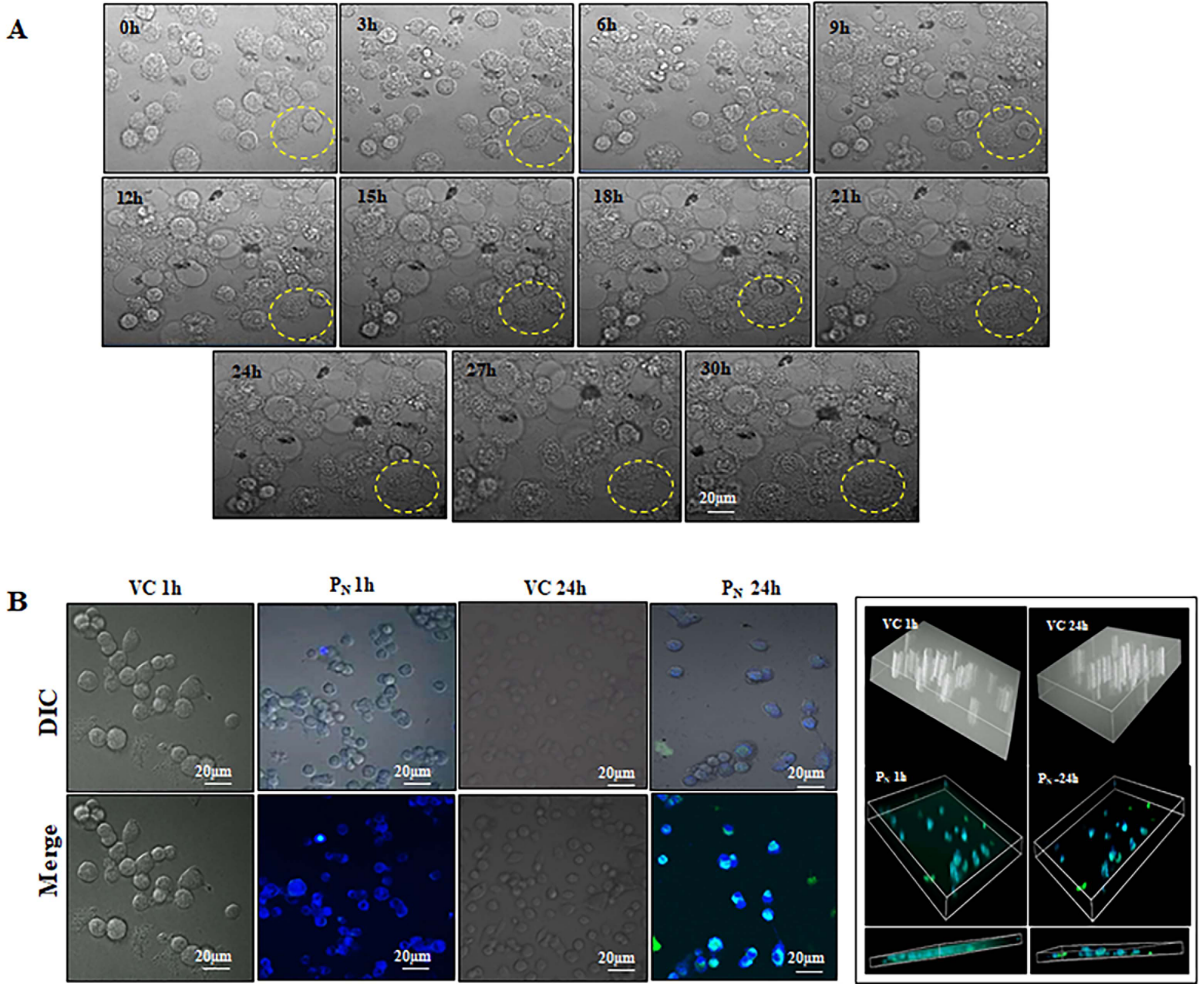
(A) Morphology of HeLa cells were appeared to be modified by P_N_ treatment such as increase cytoplasmic volume and blebbing in cell membrane that indicates initiation of apoptosis. HeLa cells was examined under live cell confocal microscope from at 0h and 30 h (B) P_N_ (100μM) easily internalized into HeLa cells within 1 h and visualized by the presence of its blue–green fluorescence after excitation at 280 nm. Z sectioning of cells exhibits P_N_ internal location and retention time period. VC, Vehicle control; P_N_, Pinostrobin.

The cells undergoing apoptosis show DNA fragmentation, chromatin condensation, cell blebbing and increased cytoplasmic volume. Live cell imaging was performed to examine the progression of cell death following P_N_ treatment up to 30 h (Fig 3A). As shown in the Fig 3A, effect of P_N_ became apparent soon after treatment as evident from time dependent deformation of cellular architecture and cell morphology that ultimately resulted in cell death. An increase in the cytoplasmic volume and blebbing could be clearly seen in P_N_-treated cell (S2 Fig, S1 Video).

Since longer cellular retention of the test molecule is not desirable, P_N_ internalization and retention in HeLa cells was also assessed using its fluorescence properties (emission of green and blue fluorescence after excitation 280 nm) (Fig 3B). Short term analysis revealed that P_N_ was able to internalize into the cells within 5 minutes of treatment and was present till 30 minutes (experimental period). No fluorescence was detected in the vehicle treated cells (S3 Fig). Subsequently, P_N_ retention was followed for a period of 24 h. As evident from the Fig 3B, significant presence of P_N_ was seen within the cells at 1 h post-treatment, which was significantly reduced at 24h in the treated cells. These changes were further confirmed by Z sectioning analysis of the treated cells (Fig 3B).

### P_N_ modulates ROS levels

It is well known that apoptosis is induced by ROS generation and depletion of intracellular antioxidant [27], we investigated P_N_-upregulated ROS production in HeLa cells using DCFH-DA dye. Presence of higher fluorescence events further confirmed the production of ROS by P_N_. P_N_ treatment (100 μM) resulted in significantly increased ROS levels (*p≤0*.*001*) when compared to vehicle treated cells (Table 1, S4 Fig).

**Table 1:** Changes in antioxidant status.

Groups	GSH (μg/mg protein)	NO_2_^-^ (μg/mg protein)	ROS (MFI)
**VC**	4.11±0.08	7.98±0.51	12.14±1.49
**P**_**N**_ **(50 μM)**	3.65±0.29^a^	5.83±0.29^c^	32.27±6.67^b^
**P**_**N**_ **(100 μM)**	2.84±0.47^b^	4.85±0.64^c^	54.20±6.73^d^
**D**_**X**_ **(10 μM)**	3.76±0.11	5.37±0.158^c^	43.69±6.12^d^

**Note:** Data represent mean±SD of three individual experiments. P_N_-treated groups were compared with VC group. Results were considered significant (two way ANOVA, Dunette,s multiple comparison test) in comparison to vehicle control (VC) group. Significance difference (p value) of treated group is calculated with respect to vehicle treated group.

^a^, *p≤*0.*05*

^b^, *p≤0*.*01*

^c^, *p≤0*.*005*

^d^, *p≤0*.*001*

Since DCFH-DA staining clearly indicated increased ROS production, intracellular nitrite and GSH levels were also measured in the P_N_-treated cells as their levels also indicate the redox state of the cell [26, 27]. Reduction (1.4±0.4 fold as compared to vehicle treated group) in GSH levels in P_N_-treated cells was comparable to that observed with vehicle control. However, 3.1±0.6 fold decreases was observed in NO_2_^-^ levels in P_N_-treated cells when compared to vehicle treated group. Elevated ROS levels in P_N_-treated cells also in line with diminished mitochondrial membrane potential, confirmed the induction of apoptosis by P_N_. Thus, the observed reduction in GSH and NO_2_^-^ levels with extensive increase in ROS together is an interesting finding that could be one of the causes of apoptosis initiation in P_N_-treated cells.

### P_N_ promotes externalization of phosphatidyl serine and DNA damage

**Fig 4:**
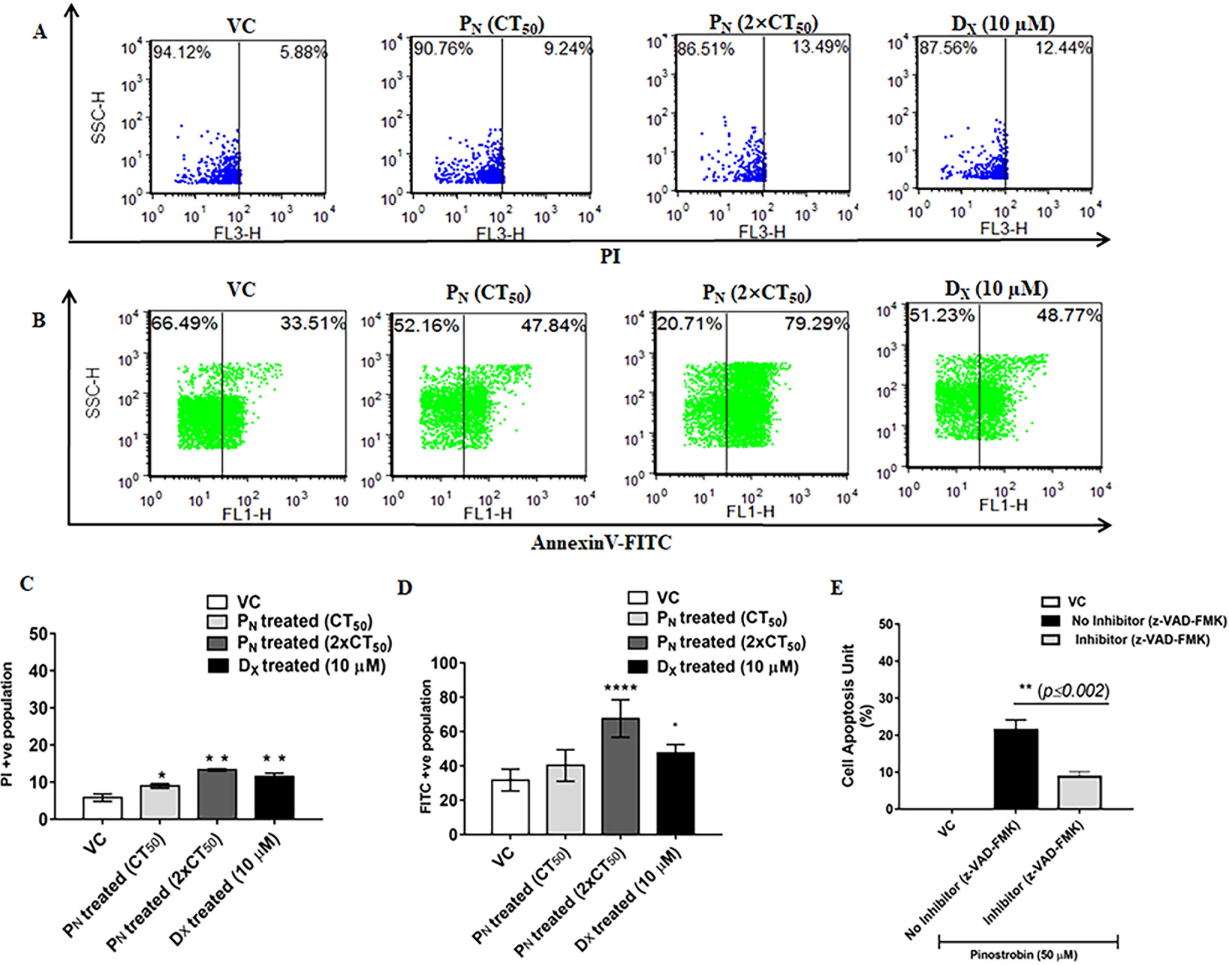
P_N_ elevates Apoptosis and DNA damage: (A) Representative dot-plot of flow-cytometric analysis of P_N_ treated, vehicle treated and D_X_ treated HeLa cells labeled with PI and analyzed by FCS Express.v5 software. (B) Representative dot-plot of flow-cytometric analysis of P_N_ treated, vehicle treated and D_X_ treated HeLa cells labeled with AnnexinV-FITC and analyzed by FCS Express.v5 software. (C) Changes in mean fluorescence intensity (PI) after P_N_ treatment in HeLa cells. (D) Changes in mean fluorescence intensity (AnnexinV-FITC) after P_N_ treatment in HeLa cells. Ordinary one-way ANOVA (Dunnett's multiple comparisons test) was performed to calculate the statistical difference (*p*≤0.05) among all treated groups as compared to vehicle treated group. (E) Changes in apoptotic unit (%) were measured via tunnel assay on incubating P_N_ treated HeLa cells with Caspase 3 inhibitor (Z-DEVD-FMK-Caspase-3. Student t-test (two-tailed, paired) was performed to calculate the statistical difference (*p≤0*.*002*) among all treated groups as compared to vehicle treated group. P_N:_ Pinostrobin, D_X:_ Doxorubicin, VC: Vehicle control. *, *p≤*0.05; **, *p≤*0.01; ***, *p≤*0.005; ****, *p≤*0.001.

Microscopic examination and live cell imaging indicated apoptotic mode of cell death in P_N_-treated cells. Apoptotic mode of cell death by P_N_ administration in HeLa cells was further confirmed by annexinV-FITC and PI staining [10]. Translocation of phosphotidylserine (PS) from the inner side of plasma membrane to the cell surface allows annexin V binding to PS in early apoptotic cells. Subsequently membrane integrity is compromised allowing uptake of PI and its binding to the cellular DNA or RNA during late apoptosis [10]. An increase (8.87±1.8%, *p≤0*.*01;* Fig 4A & 4C) in necrotic cell population was observed in P_N_-treated cells, however, P_N_ treatment resulted in significant increase in apoptotic cell population rate (67.62±10.94%, *p≤0*.*001*; Fig 4B & 4D) of early apoptotic cells was observed as compared to vehicle treated cells. To confirm that P_N_ treatment indeed caused apoptosis, TUNEL assay was performed to detect DNA fragmentation and for identification of cells undergoing apoptosis, in the presence or absence of apoptosis inhibitor. In the absence of apoptosis inhibitor, P_N_ treatment showed significantly increase of 21.40±2.71% (*p≤0*.*005*) in cell apoptosis unit when compared to vehicle controls (Fig 4E). Presence of apoptosis inhibitor in P_N_-treated cells caused a reduction in cell apoptotic units. These findings confirmed P_N_ induced apoptosis mediated cell death.

### P_N_ promotes mitochondrial damage and nuclear condensation

**Fig 5:**
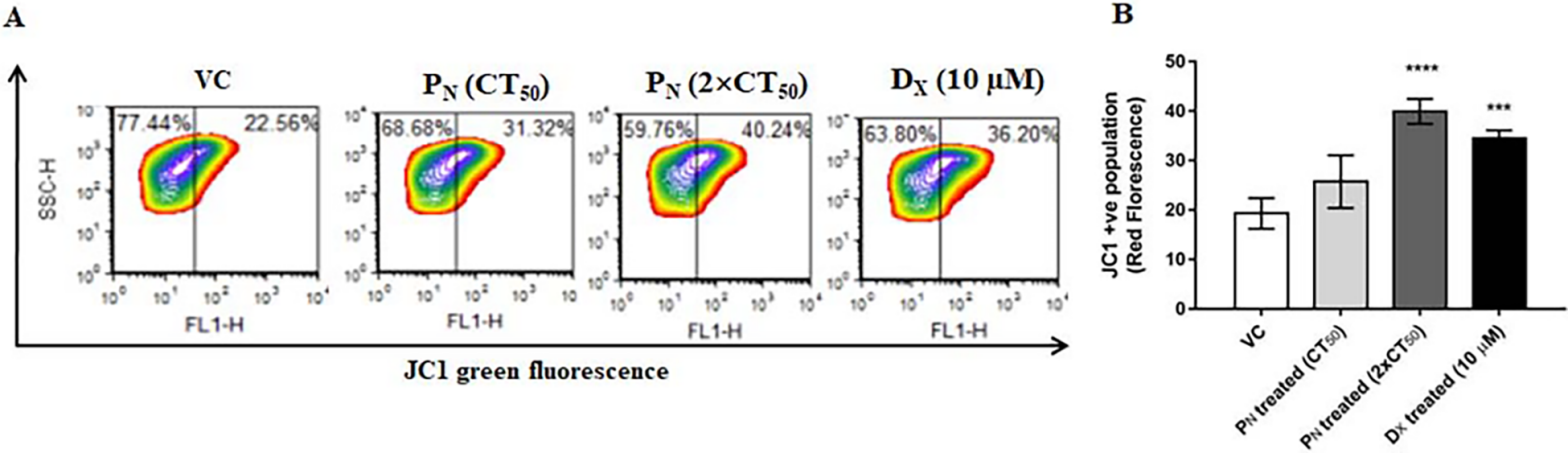
Effect of P_N_ administration on the mitochondrial membrane potential (*ΔΨm*) in HeLa cells assayed for depolarized mitochondria (i.e. mitochondria having low membrane potential). (B) Changes in *ΔΨm* were observed using mitochondria specific fluorescent probe JC-1. Monomeric green fluorescence increased as *ΔΨm* level decrease. In dot-plot, X-axis represents mitochondria with low membrane potential (Green fluorescence). Ordinary one-way ANOVA (Dunnett's multiple comparisons test) was performed to calculate the statistical difference (*p≤ 0*.*05*) among all treated groups as compared to vehicle treated group.

Mitochondrial membrane potential (*ΔΨm*) is an indicator of cellular health. It is one of the major marker of mitochondrial membrane integrity and a decrease in *ΔΨm* is one of the early events that lead to apoptosis [32]. JC-1 forms aggregates (red fluorescence) in mitochondria of live cells whereas in apoptotic cells due to poor health of the mitochondria and loss of potential, these aggregates leak out from the mitochondria to the cytosol as monomers (green fluorescence) [9]. To assess if P_N_ treatment reduced the *ΔΨm* in HeLa cells, staining of P_N_-treated cells with JC-1 dye was performed. P_N_-treated cells showed a significant increase in JC-1 monomers as evident from higher green fluorescence suggesting reduction in mitochondria membrane potential (*ΔΨm*) (Fig 5). On the other hand, intense red fluorescence of JC-1 aggregates in the vehicle treated cells indicated high *ΔΨm*. Thus, an increase in JC-1 green to red fluorescence ratio in the P_N_-treated cells is consistent with the TEM results (Fig 2C) wherein mitochondrial membrane disintegration was observed in P_N_-treated cells, which could possibly be caused due to evident loss of *ΔΨm*. Quantitative analysis of dissipation of mitochondrial membrane potential using JC-1 probes by flow cytometry further confirmed reduction in mitochondrial membrane potential (Fig 5). P_N_-treated (100 **μ**M) cells showed significant increment of 20.67±4.6% *(p≤0*.*001*) JC-1 monomer fluorescence events at 48h with respect to vehicle control which was comparable to the observed increase (15.19±2.1%) in the D_X_ treated cells (positive control).

**Fig 6:**
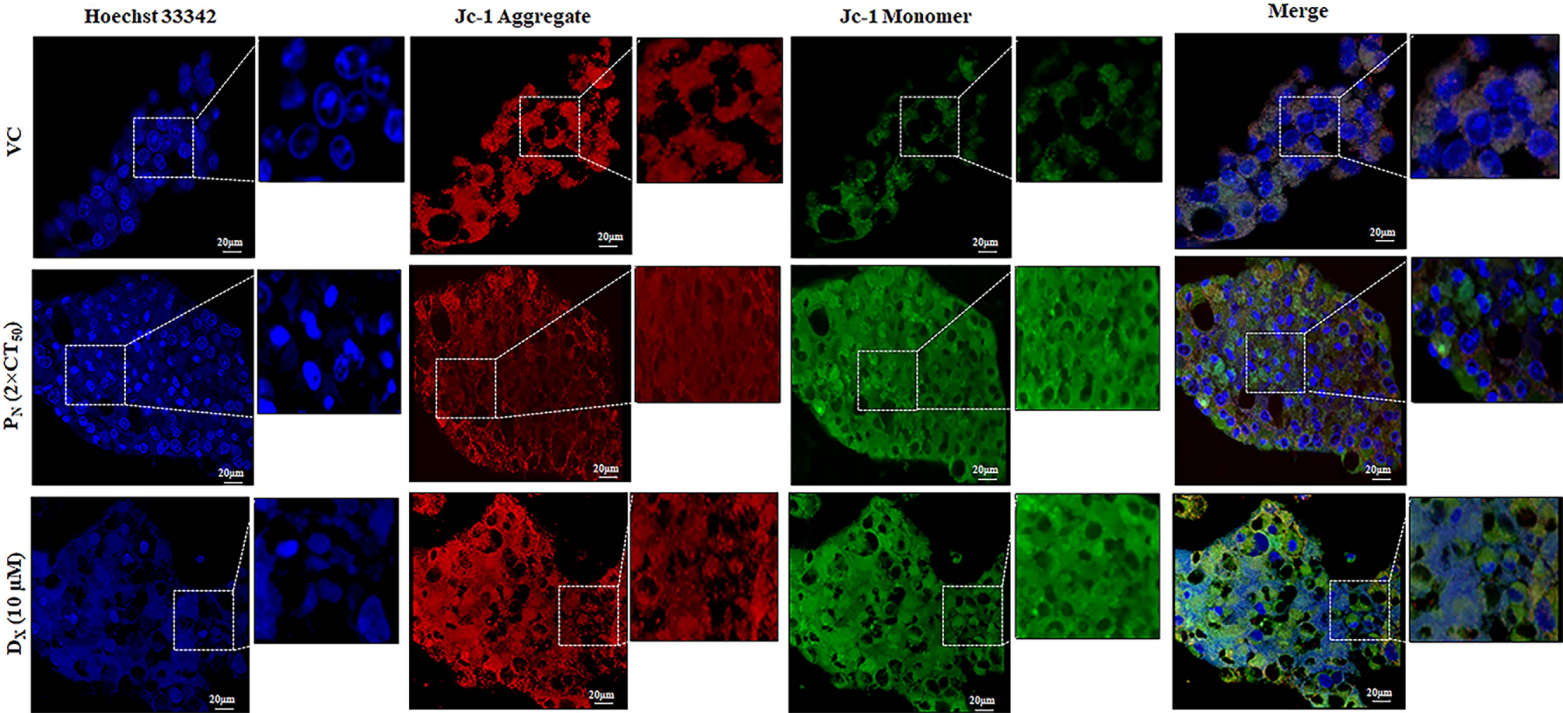
Fluorographic representations of HeLa cells stained with Hoechst 33258 and JC-1dyes visualized at 40× magnifications to observe mitochondrial depolarization and chromatin condensation in the nucleus, respectively. P_N:_ Pinostrobin, D_X:_ Doxorubicin, VC: Vehicle.

To examine the chromatin condensation and deformation of nuclear structure upon P_N_ treatment, the cells were stained with Hoechst 33342 stain, that emits blue fluorescence when bound to double stranded DNA and distinguishes apoptotic cells with condensed chromatin from normal cells. After 48h P_N_ and D_X_ exposure to HeLa cell showed remarkable changes such as bright, condensed and fragmented nuclei at 40× magnification and under live confocal microscopy (Fig 6), while vehicle treated cells showed dim and dark blue color of nucleus with intact nuclear membrane. Thus the rate of JC-1 monomer and nucleus degradation was increased in time dependent manner. The findings suggest that P_N_ has potential to target both mitochondria and nucleus simultaneously, and inhibits its regulatory functions to mediate cell death by activation of intrinsic apoptosis pathway.

### Effect of P_N_ on the cell cycle progression

**Fig 7:**
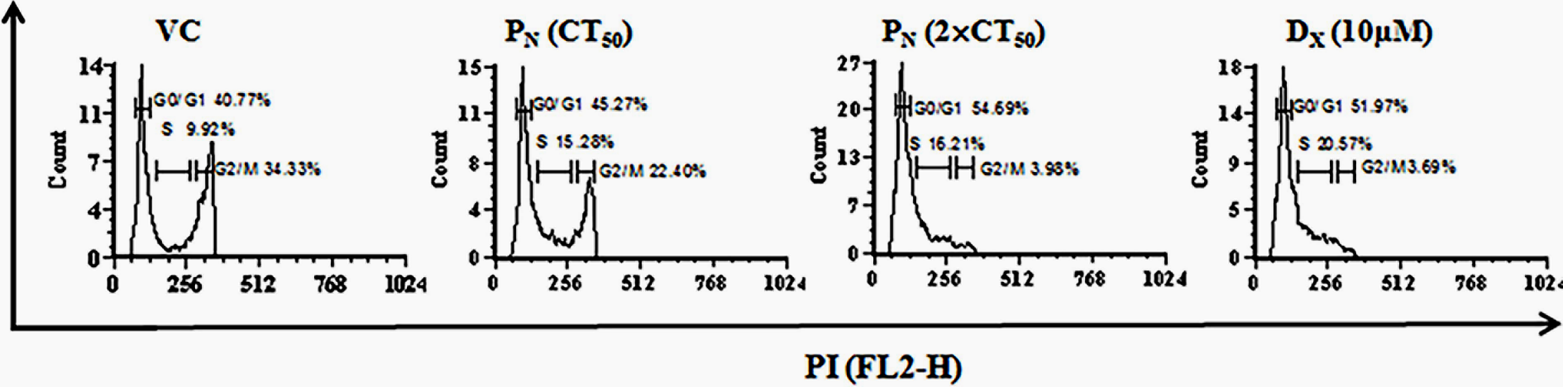
Cell cycle analysis of apoptotic HeLa cells was performed by flow cytometry after propidium iodide staining. G1/S phase of cell cycle was found arrested by pinostrobin treatment at 48 h. The X-axis (FL-2H) represents DNA content and Y-axis (counts) shows the number of cells in each phase of cell cycle.

Deregulation of the cell cycle is one of the major biomarker illustrating the interactions between proteins that process incoming internal and external signals for whether the cell proliferates or differentiates [33]. DNA fragmentation induced by P_N_ is likely to interrupt the cell cycle progression at cell cycle checkpoints ultimately leading to cell death. Therefore, we studied the changes in cell cycle after P_N_ treatment (50 μM & 100 μM) using flow cytometry at 48 h post-treatment. P_N_-treatment resulted in cell cycle arrest at G1/S phase (Fig 7). An increase in number of G1 cell population in P_N_-treated cell (54.69+ 3.43% with 100 μM) compared to vehicle control (40.77+1.22%) was observed. Similarly, the positive control D_X_ treatment also resulted in an increase in number of cell population (51.97+2.25%) in G1/S phase in comparison to vehicle control.

### Changes in apoptotic proteins on P_N_ exposure

**Fig 8:**
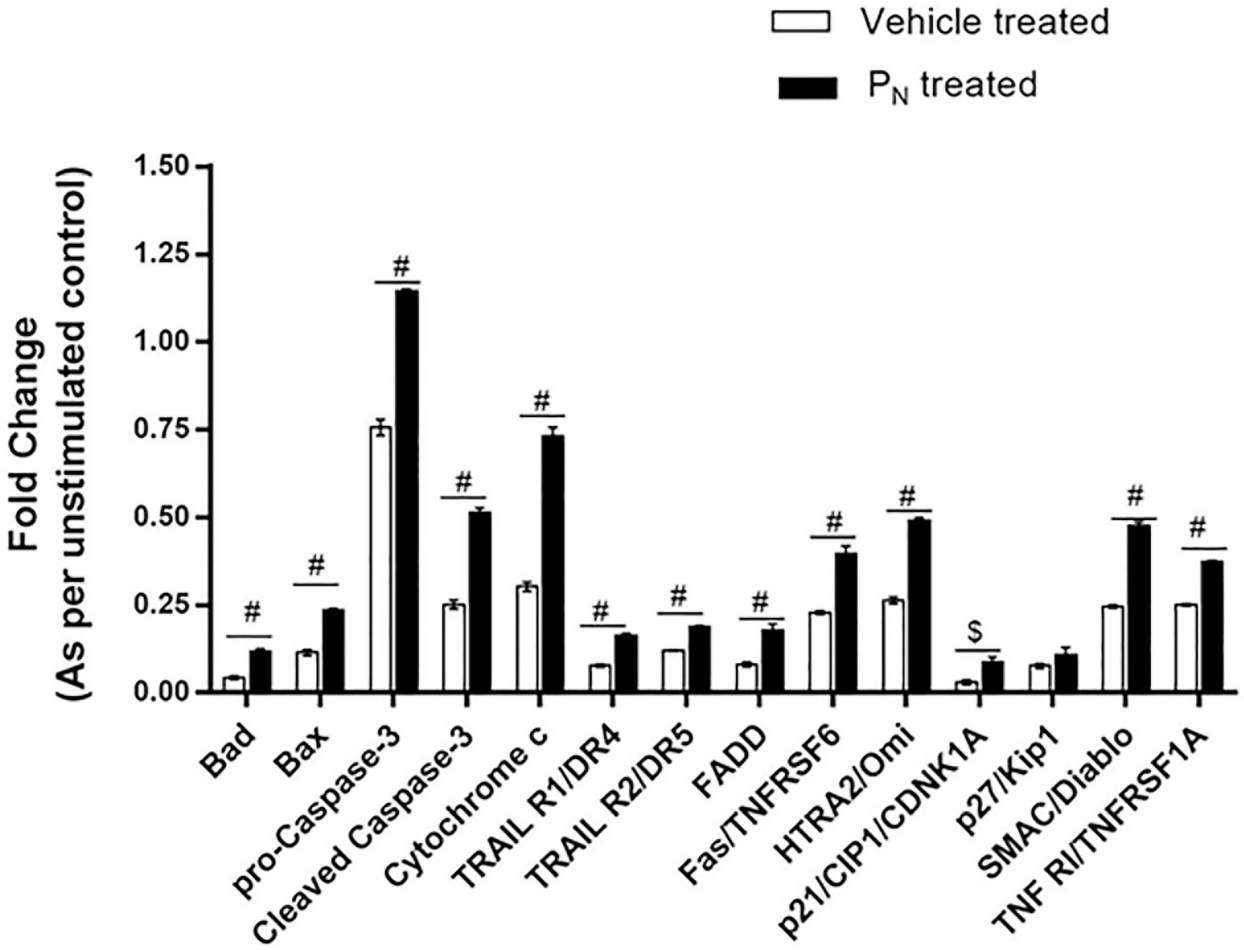
Proteome profiling of apoptosis associated proteins. Relative levels of different proteins in the cell lysates from vehicle treated (VC) and P_N_ treated cells harvested 48 h post-treatment were determined using proteome profiler array kit from R&D Systems. Protein expressions showing significant changes are depicted graphically. Ordinary two-way ANOVA (Bonferroni's multiple comparisons test) was performed to calculate the statistical difference among all treated groups as compared to vehicle treated group. $ represents *p≤0*.*001* and # *p≤0*.*0001*.

In order to identify the mechanistic pathway (extrinsic and intrinsic) by which P_N_ induced apoptosis, expression analysis of anti- and pro-apoptotic proteins in the P_N_-treated cells’ lysate was carried out using human apoptosis protein profile array at 48h post-treatment,. Our results showed that P_N_ treatment resulted in an increase in proteins involved in both the intrinsic and extrinsic pathways. Involvement of extrinsic apoptosis pathway is evidenced from higher expression level of TRAIL R1/D4, TRAIL R2/D5, TNFα, Fas and FADD proteins in P_N_-treated cells as compared to vehicle treated cells (Fig 8; S5 Fig). In addition, we observed significant increase in levels of proteins such as Bad, Bax, caspase 3, cyto-c, which were involved in early apoptosis. Interestingly, we observed slight changes in p53 (major tumor suppressor protein) expression level of P_N_-treated cells as compared to vehicle treated groups. Also, an increase in Fas/TNFRSF6, HTRA2/Omi, p21/CIP1/CDNK1A, SMAC/Diablo, TNF R1/TNFRSF1A levels, involved in intrinsic pathways were higher in P_N_-treated cells when compared to vehicle treated cells.

## Discussion

Evasion of apoptosis by malignant cells is a hallmark of cancer and induction of apoptosis by the cytotoxic anticancer agents is one of the strategies adopted for developing chemotherapeutics for the treatment of cancer [34, 35]. Experimental evidence in the last two decades indicates that dietary constituents, particularly medicinal plant derived constituents possess capacity to control the complex phenomenon of carcinogenesis through alteration of gene expression and induction of apoptosis [36, 37]. Medicinal plants derived products such as flavonoids, have gained attention in recent years as a new alternative approach to selectively induce apoptosis in cancer cells. Several studies have been conducted on partially purified plant extracts and also on pure constituents possessing anti-proliferative properties that can be further exploited as potent anticancer drugs. A wide variety of flavonoids derived from medicinal plants have been shown to confer potent anti-mutagenic and anti-carcinogenic activities both *in vitro* and *in vivo* [36, 37]. Xiao and his coworkers (2011) demonstrated inhibition of gastric cancer cells proliferation *in vitro* by LicochalconeA, isolated from *Glycyrrhiza glabra* [38]. On the other hand, another flavonoid Glabridin isolated from the *Glycyrrhiza glabra* inhibited cell metastasis, decreased tumor angiogenesis and inhibited invasion of MDA-MB-231 cells [39]. Baicalein and Baicalein, flavones present in number of plants, have been reported to exhibit anti-proliferative and anti-tumor activity in different types of cancers [40, 41]. Likewise, Quercetin, another plant-derived flavonoid inhibited growth and induction apoptosis in several types of tumor cells, such as, human cervical cancer, prostate cancer, oral cancer, osteosarcoma, etc [40, 42, 43]. Thus, some flavonoids show selective growth inhibition of certain types of cells whereas some show broad spectrum activity. Many of the flavonoids show synergistic action in different cancer cells [43, 44]. Therefore, systematic studies are required for the development of target oriented therapeutics from natural products with broader spectrum.

The reduction of cancer growth by different flavonoids could occur due to suppression, blockage, and inactivation of various signaling molecules during apoptosis. However, exact mechanisms by which many of the flavonoids exert their anticancer activity are yet to be elucidated.

P_N_ is a naturally occurring compound present in honey and various plants that are consumed as diet supplements. Various pharmacological activities of different plants have been attributed to P_N_. Anticancer activity of P_N_ has also been reported *in vitro* in few cell lines [45–47]. Moderate cytotoxicity (20%) by P_N_ at 50 μM was demonstrated in HepG2 cells line [46]. Ashidi and coworkers isolated all the major constituents of *Cajanus cajan* including P_N_ and demonstrated their cytotoxicity in different cancer cell lines [45]. Of various active constituents isolated from this plant, P_N_ exhibited maximum dose-dependent toxicity on the CCRF-CEM cells derived from acute T-lymphoblastic leukemia patient at a CT_50_ of ~10 μM, whereas in other cell lines of breast cancer MCF-7, lung cancer COR-L23 and melanoma (C32) origin, the IC50 was determined to be ~100 μM [44, 45]. In HepG2 cells of liver cancer origin, P_N_ isolated from leaves of *Carya cathayensis* showed cytotoxic effect with a CT_50_ of at >100 μM against HeLa and HepG2 cells [47]. Thus, P_N_ showed broad spectrum differential cytotoxicity in different cell lines. While these workers reported anti-proliferative activity of P_N_ in a variety of cells, its mechanism of action was not elucidated. In the present study, we observed P_N_-induced ROS generation and toxicity in different cervical cancer cells with minimal effect on non-cancerous HEK cells at 48h post-treatment (S1 Fig). Of the three cervical cancer cell lines HeLa, SiHa and Ca Ski, maximum cytotoxicity was observed in HeLa cells (minimum CT_50_). Therefore, HeLa cells were used for further investigations to understand its mode of action.

Abdelwaheb *et al*., (2011) claimed P_N_, as the main active constituent of methanolic extracts of *Boesenbergia rotunda* exhibited anti-ulcer activity [48], which could be attributed to indirect anti-oxidant mechanism of P_N_, but not to the intervention with nitric oxide and COX inflammation pathways. Yet, another investigation demonstrated that P_N_ inhibited voltage-gated sodium channels of mammalian brain [49]. High concentrations of P_N_ could control the change of the membrane potential as they remained less negative under high concentrations. Based on these reports, P_N_ shows promise as a potential anti-cancer molecule.

In the present study, we used both morphological and biochemical characterization of the P_N_-treated cells to decipher its possible mechanism of action. As evident from our results, P_N_ demonstrated dose dependent growth inhibition on different types of cancer cell lines *in vitro* without showing any adverse effect on non-cancerous cells. HeLa cells were found to be relatively more sensitive cells with CT_50_ values at 50 μM, respectively when compared to Ca Ski and SiHa with CT_50_ values at 75 and 100 μM, with no toxicity to non-cancerous cells. The cytotoxic dose of P_N_ for these cancer cell lines is much lower than that reported by other investigators [47]. Though P_N_ showed higher CT_50_ values in comparison to other currently available chemotherapeutics it shows negligible cytotoxicity in normal cells even at 4×CT_50_ concentration (S1 Fig). Thus, preliminary cell proliferation experiment clearly demonstrated that P_N_ was cytotoxic to cancer cells only. Since, P_N_ is a constituent of many foods consumed daily; its effective higher concentrations are not likely to have adverse effect. Several other flavonoids have also been shown to be effective in similar concentration range in different types of cancerous cell lines [40, 50]. CT_50_ of Quercetin ranged between 70–350 μM in prostate, uterine, breast, leukemic and bone cancer cell lines [40, 50]. Similarly, Kaempferol and Rutin found to be efficient all together from 50–100 μM and 100–200 μM doses respectively in different cell lines [40]. Pharmacokinetics studies demonstrated that P_N_ and other similar flavonoids have short shelf-life (6 h) and low bioavailability [51]. Flavonoids are usually ingested with other foods components, resulting in the complexation or precipitation of flavonoid compounds, thus limiting their absorption and bioavailability [51]. Recently, new strategies are being developed for promotion of flavonoids by the pharmaceutical industries such as use of enhancers, modification in structure of parent flavonoid to produce novel derivative [51, 52], thus reducing the effective dose.

For a therapeutic agent to be acceptable and effective, it is desired that the molecule is taken up by the cell and cleared from the system effectively. A number of studies have been reported to explain the self-internalizing ability of therapeutic molecules which is imperative to understand their mechanisms of entry. We used innate fluorescence property of P_N_ (emitting green and blue fluorescence at excitation 280 nm) to examine its entry and retention in HeLa cells by using live cell imaging and Z sectioning of the treated cells using live cell confocal microscope. This allowed us to monitor its entry and retention in real-time period at unicellular level. Similarly, earlier live cell imaging has been employed to examine the delivery of therapeutics using cell-penetrating peptides [24]. Z sectioning data clearly demonstrated that P_N_ entered into the cells as early as 5 min of the addition, with a time dependent increase in intracellular P_N_ fluorescence till 30 min. A significant decrease in the fluorescence at 24 h post-treatment indicated fast and effective clearance of P_N_ from the cells. This property makes it more acceptable than those drugs which have longer retention time and result in accumulation in the system, thus causing undesirable adverse effects. After exposure of P_N_, the cell lost their membrane integrity and blebbed which are typical signs of apoptosis. Microscopic examination revealed that both P_N_ and D_X_-treated cancer cells showed typical morphological features of apoptosis such as reduction in cell volume, cell shrinkage, low confluency of cell, nucleus condensation, DNA fragmentation, alteration in membrane symmetry, membrane blebbing and apoptotic bodies formation.

The primary goal of cancer chemotherapy is to specifically target cancer cells without any or minimal effect on normal cells. However, current anticancer drugs fail to meet these criteria. Cancer cell-selectivity is the most important criteria in the development of an anti-cancer drug. Lack of toxicity without any changes in normal cell structure and architecture by P_N_ observed in the present study is an early indication that it may possess a very good safety profile. P_N_-induced apoptosis, indicated by morphological examination of the cells, was further confirmed by annexinV-FITC/PI staining and TUNNEL assay wherein a significant increase in early apoptotic cells was observed in the treated cells. An increase in the number of PI stained nuclei was also observed in P_N_-treated cells. These data together with annexinV-FITC stained cells clearly confirm apoptotic cell death in P_N_-treated cells.

Our results using AO/EB staining showed condensed and fragmented nuclei, which are also supported by the findings of Yong and Abd Malek [25]. These investigators also demonstrated the presence of condensed and fragmented nuclei through Hoechst 33342/PI staining in the Ca Ski cells treated with xanthohumol (another flavonoid). These results suggested that P_N_ did affect cell membrane architecture and its integrity could ultimately lead to cell death. Similar results have been obtained with another dietary flavonoid, fisetin, treatment with which resulted in loss of membrane integrity in osteocarcinoma cells [53].

Role of mitochondria in apoptosis is well established and disruption of active mitochondrial function is one of the earliest signature events that occur during early stages of apoptosis. Earlier studies by Yong and Malek have demonstrated that xanthohumol, a phytochemical present in female flowers of *Humulus lupulus* and its flavanone derivatives induced DNA fragmentation, cell cycle arrest at S phase and inhibited growth of Ca Ski cervical cancer cells through induction of apoptosis [25]. Other natural compounds, particularly polyphenolic flavonoids and certain alkaloids have also been reported to induce cell death through apoptosis process in malignant cancer cells [54, 55].

In line with apparent morphological changes induced by P_N_, TUNEL assay also showed DNA fragmentation in P_N_-treated cells. Absence of any or negligible fragmentation in control cells clearly indicated that the event is specifically induced by P_N_. Significant reduction of DNA fragmentation in the P_N_-treated cells in the presence of apoptosis inhibitor (Z-DEVD-FMK-Caspase-3 inhibitor) further confirmed apoptosis inducing potential of P_N_.

ROS-mediated apoptotic signaling is associated with decreased cellular GSH levels and the loss of cellular redox balance. Decrease in GSH levels can occur through ROS-induced GSH oxidation from the cells; the resultant GSH reduction would increase further ROS production during oxidative challenge [55, 56]. Many chemotherapeutic agents exert their anti-cancer effect through ROS generation. Therefore, we assessed if P_N_ could induce ROS production in cancer cells. High ROS production in P_N_-treated cells correlates well with the decreased mitochondrial membrane potential and subsequent increase in apoptosis. Li and his coworkers have also demonstrated an increase in ROS levels by fisetin leading to apoptosis in osteocarcinoma cells [53]. Similarly, Zhang *et al*, have demonstrated isoliensinine induced apoptosis in triple-negative human breast cancer cells through ROS generation [56]. Thus, our results on P_N_-induced ROS generation and oxidative stress are in line with these reports for induction of apoptosis by damaging mitochondrial membrane.

In addition to ROS production, depletion of intracellular antioxidant enzymes has also been attributed for apoptosis induction. In the present study, intracellular GSH was also found to be significantly reduced in P_N_-treated cell when compared to vehicle treated cells. This depletion of GSH could be due to higher production of ROS. Further, GSH plays major role in protection against oxidative stress. Thus, depletion of GSH could enhance the susceptibility of oxidative stress caused by ROS, ultimately facilitating cell death. Reduction in endogenous NO, that plays an important role in tumor progression, angiogenesis and metastasis, at different time P_N_ post-treatment further demonstrate anti-tumorigenic effect of P_N_. Vijaya Padma *et al*., (2007) have also reported reduction of NO and induction of apoptosis in HepG2 cells after exposure to ginger extract [27].

During mitochondria mediated apoptosis, disturbances in mitochondrial functions lead to activation of caspase-9, reduction in its membrane potential and cleavage of Bcl-2 protein. Our initial findings on induction of apoptosis by ROS production and disruption of mitochondrial membrane potential prompted us to assess activation of caspase cascade and analyses possible pathway by expression analysis of different proteins involved using human apoptosis protein profile array. Increased expression of Smac/DIABLO, HtrA2/Omi and cytochrome c expression levels in P_N_-treated cells were observed when compared to vehicle treated cells. These mitochondrial proteins [Smac/DIABLO, HtrA2/Omi and cytochrome c] are released from mitochondria to cytosol in response to stress and induce apoptosis. Released cytochrome c forms a complex with Apaf-1 and caspase-9 to activate caspase cascade in the presence of ATP and trigger apoptosis. Activation of caspase 9 and caspase 8 by P_N_ suggests that both extrinsic and intrinsic pathways are involved that ultimately lead to activation of the key executional caspase, caspase-3. Thus, an increase in expression of these proteins reconfirms apoptosis inducing potential of P_N_. Activation of caspase 3 has also been reported by other flavonoids that caused cell death in cancer cells. In addition, levels of some other pro-apoptotic BCl-2 family proteins such as Bad and Bax were also found to be elevated and highly expressed in P_N_-treated cells. These proteins form pores in the outer mitochondrial membrane and stimulate apoptosis, while the anti-apoptotic proteins including Bcl-2 and Bcl-xL inhibit pore formation. Jürgensmeier *et al*, have reported the overexpression of bax accountable for the release of cytochrome c [57]. Thus, the expression analysis of different proteins involved in apoptosis indicated that P_N_ induced apoptosis in the tested cancer cells not only by activation of caspases but also by modulating the expression of apoptotic proteins. Such induction of apoptosis through mitochondrial as well as intrinsic pathway has also been reported for other flavonoids and resveratrol [54, 58].

Expression analysis also indicated involvement of extrinsic pathway of apoptosis in P_N_-treated cells as increased levels of TRAIL R1/D4, TRAIL R2/D5, TNFα, Fas and FADD were observed in some of the P_N_-treated cancer cells at 48 h in comparison to vehicle treated cells. Earlier studies have established that TNF (Apo-2L) binds to death receptors DRs (4 and 5) and induces transducing death signal for cell death [59]. Likewise, we also observed an increase in TNFR1 and Fas at 48 h in the treated cells. Increased TNF levels are likely to effectively bind to TRAIL-R1 and TRAIL-R2 and induce cell death. Further, P_N_-treated cancer cells also showed an upregulation of p53 with observed increase in the expression of p21 and p27. These results suggest that P_N_ could have arrested cell cycle progression which is evidenced by cell cycle arrest in G1/S phase in the P_N_-treated cells. An overview of the protein expression profile provides further evidence of induction of both extrinsic and intrinsic apoptosis pathways and activation of p21, p27 through p53 leading to cell cycle arrest. Thus, findings from morphological, biochemical and molecular analyses collectively suggest that P_N_-induced cell death resulted due to ROS-mediated apoptosis involving both the extrinsic and intrinsic pathways. It is likely that P_N_ upon interaction with a molecular target (yet to be identified) in the cell triggered a signaling cascade resulting in cell death. Further investigations are required to identify the molecular target of P_N_ and deciphering the signaling pathway involved in P_N_-induced ROS-mediated cell death.

## Conclusions

The current findings present a chronological sequence of events related to the effects of P_N_ on HeLa cells. In this study, we found P_N_ induced ROS mediated apoptosis involving extrinsic and intrinsic pathway in HeLa cells. These results provide an insight on the understanding of P_N_ induced cell death mechanism and the link between ROS induced apoptosis and mitochondrial membrane permeability. Our key findings explain the mechanisms by which P_N_ regulates apoptosis phenomenon *in vitro*. However, further *in vivo* and pharmacological studies would be necessary in order to confirm its practical uses in the treatment of cancer. Nevertheless, this study provides a platform to explore other molecular targets of P_N_.

## Supporting information

S1 Fig Effect of P_N_ on cell viability and ROS production in HEK cell. (A) Cytotoxicity % (CT) assessed in HEK cells on different concentrations of P_N_ treatments as determined by MTT reduction assay at different incubation period. The bar graphs represent the percentage of cytotoxicity of P_N_ in the cells. Cytotoxicity is shown as mean ± SD derived from at least three separate experiments in triplicate wells. (B) Changes in ROS levels on P_N_ exposure. The bar graphs represent the percentage of MFI (DCF) in the cells at 24 and 48 h of incubation period. MFI is shown as mean ± SD derived from at least three separate experiments in triplicate wells.
(TIF)

S2 Fig Representation of morphological changes induced by pinostrobin at different time intervals. Induction of apoptosis is examined by enhancement of cytoplasmic volume and blebbing in cell membrane.
(TIF)

S3 Fig Internalization of pinostrobin and vehicle in HeLa cells are visualized under live cell imaging confocal microscope at various time interval and analyzed its internal location and retention time by Z sectioning of cells at 40× magnifications after excitation at 280 nm. VC, Vehicle control; P_N_, Pinostrobin.
(TIF)

S4 Fig Representative dot-plot of flow-cytometric analysis of P_N_ treated, vehicle treated and D_X_ treated Hela cells at 24 h incubation for ROS levels and analyzed by FCS Express.v5 software. (TIF)

S5 Fig Dot-blot representing the effect of P_N_ treated, vehicle treated HeLa cells on apoptosis associated proteins after 48 h of incubation. (TIF)

S1 Video Visual induction of apoptosis by pinostrobin in HeLa cells. (MP4)

## Abbreviations

ΔΨm: Mitochondrial membrane potentia
DCFH-DA: 2, 7-Dichlorodihydrofluoresceindiacetate
JC-1: 5,5,6,6-Tetrachloro-1,1,3,3-tetraethylimidacarbocyanine
HPV: Human papilloma virus
Cyt *c*: cytochrome *c*
Smac: Second mitochondria-derived activator of caspases
ApoAF-1: Apoptotic protease activating factor-1
IAP: Inhibitor of apoptosis protein
FITC: Fluorescein isothiocyanate
PI: Propidium iodide
P_N_: Pinostrobin
D_X_: Doxorubicin
