# Supplementary figures and images for: Induction of apoptosis by pinostrobin in human cervical cancer cells: Possible mechanism of action
Source: PLoS One. 2018 Feb 8;13(2):e0191523. doi: 10.1371/journal.pone.0191523 (PMC5805241; doi:10.1371/journal.pone.0191523)

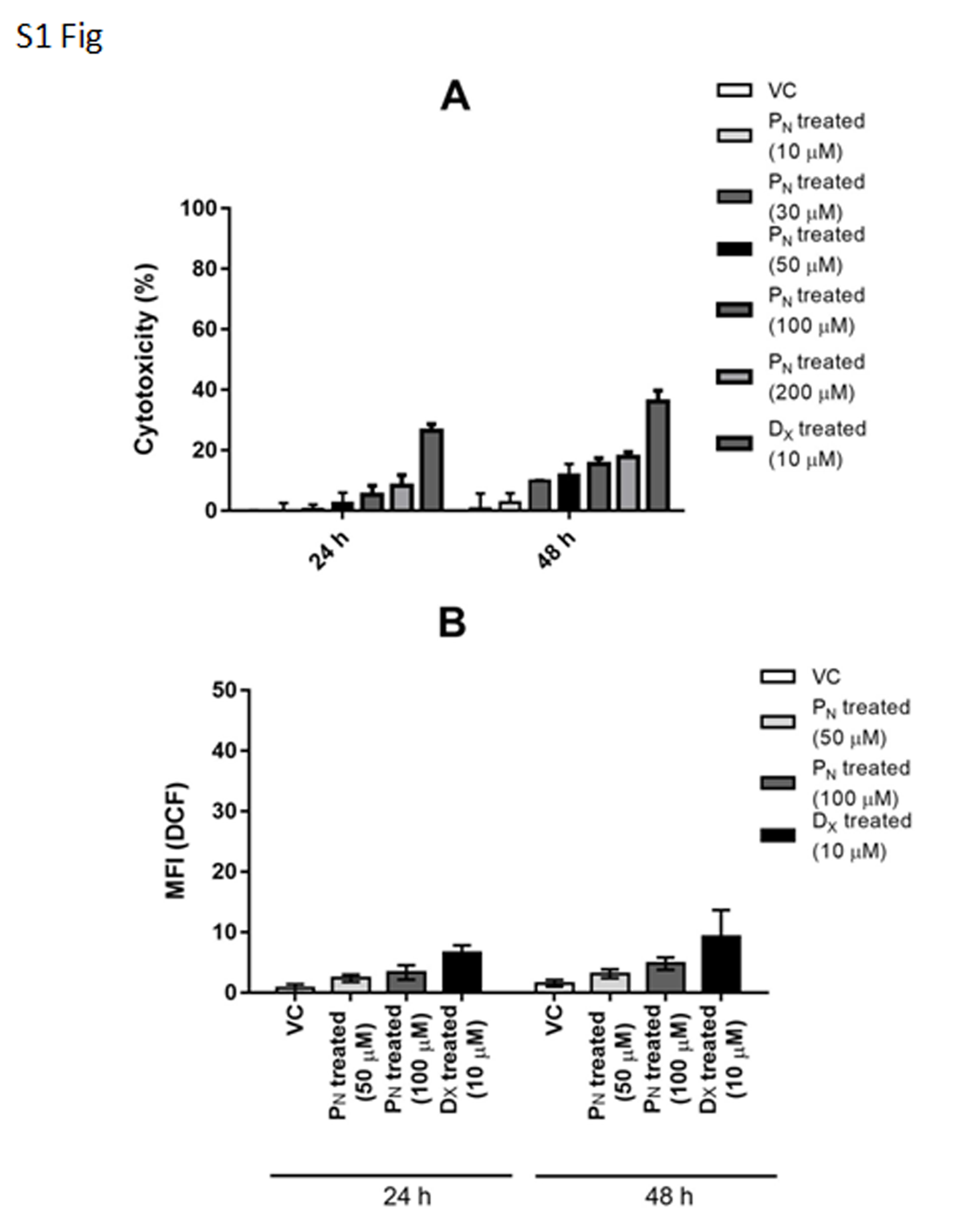

Supplement: S1 Fig — (A) Cytotoxicity % (CT) assessed in HEK cells on different concentrations of PN treatments as determined by MTT reduction assay at different incubation period. The bar graphs represent the percentage of cytotoxicity of PN in the cells. Cytotoxicity is shown as mean ± SD derived from at least three separate experiments in triplicate wells. (B) Changes in ROS levels on PN exposure. The bar graphs represent the percentage of MFI (DCF) in the cells at 24 and 48 h of incubation period. MFI is shown as mean ± SD derived from at least three separate experiments in triplicate wells. (TIF) [file pone.0191523.s001.tif]

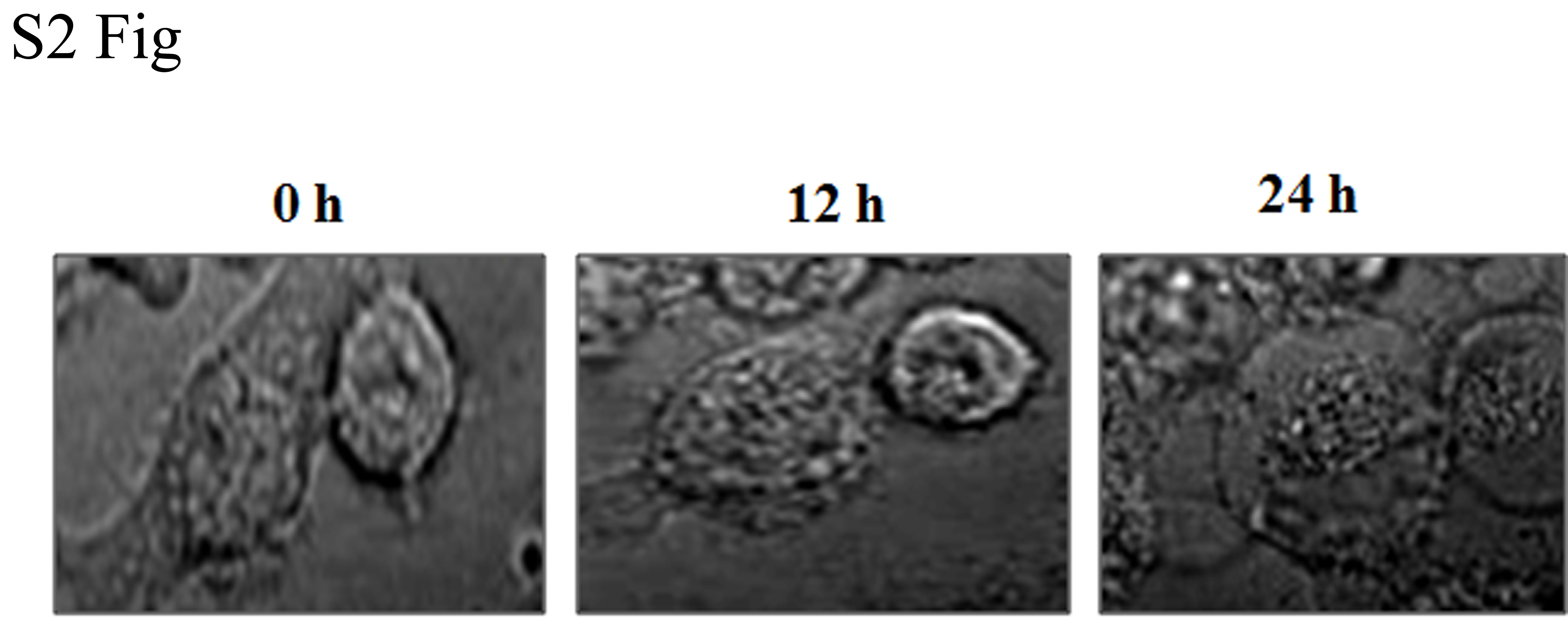

Supplement: S2 Fig — Induction of apoptosis is examined by enhancement of cytoplasmic volume and blebbing in cell membrane. (TIF) [file pone.0191523.s002.tif]

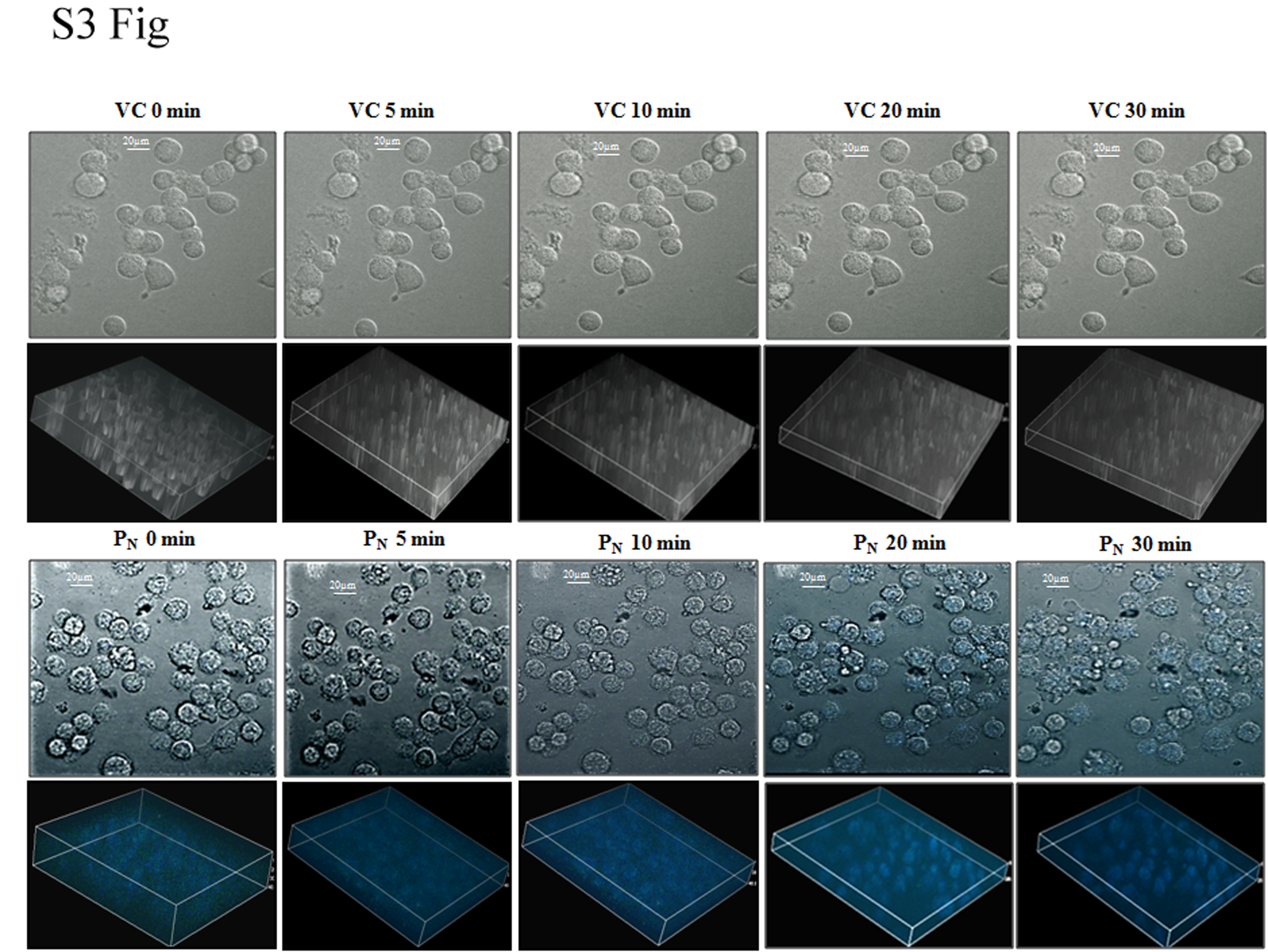

Supplement: S3 Fig — VC, Vehicle control; PN, Pinostrobin. (TIF) [file pone.0191523.s003.tif]

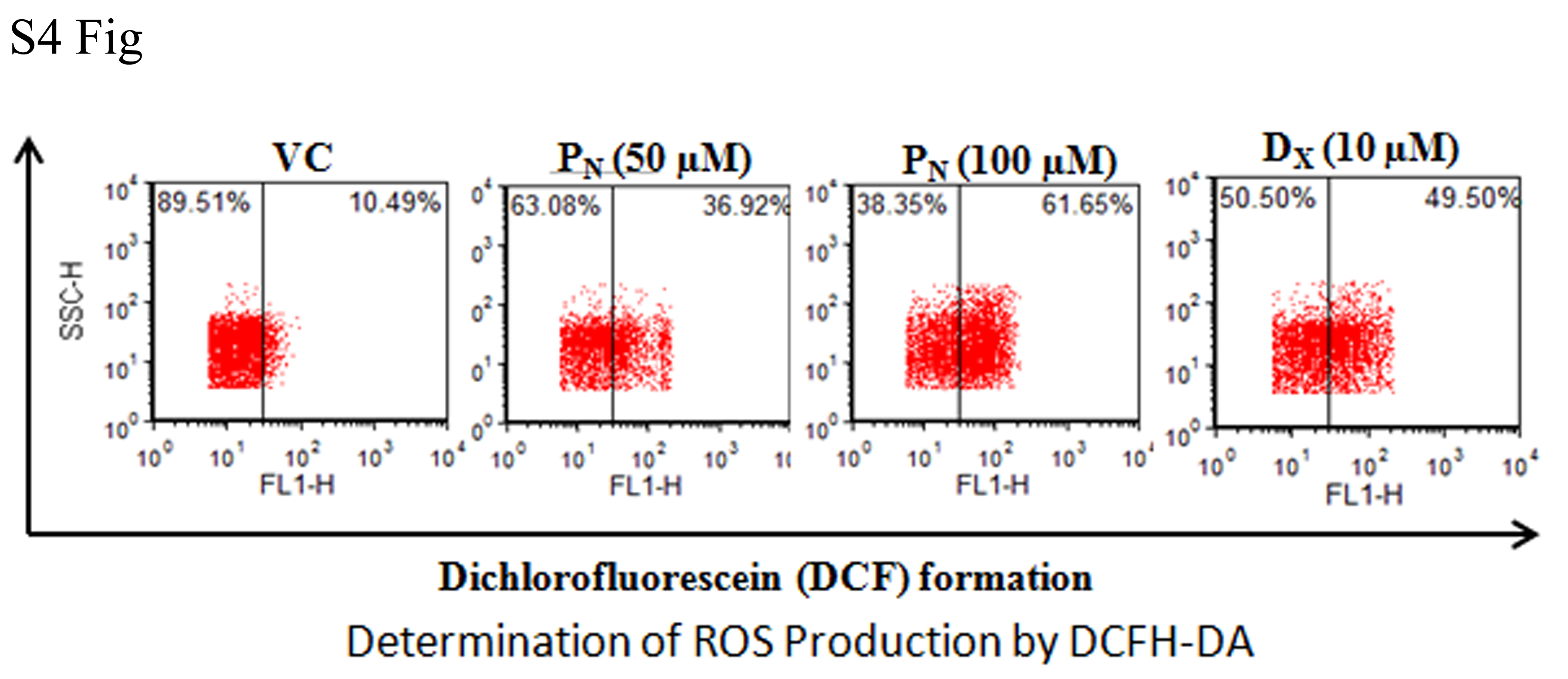

Supplement: S4 Fig — (TIF) [file pone.0191523.s004.tif]

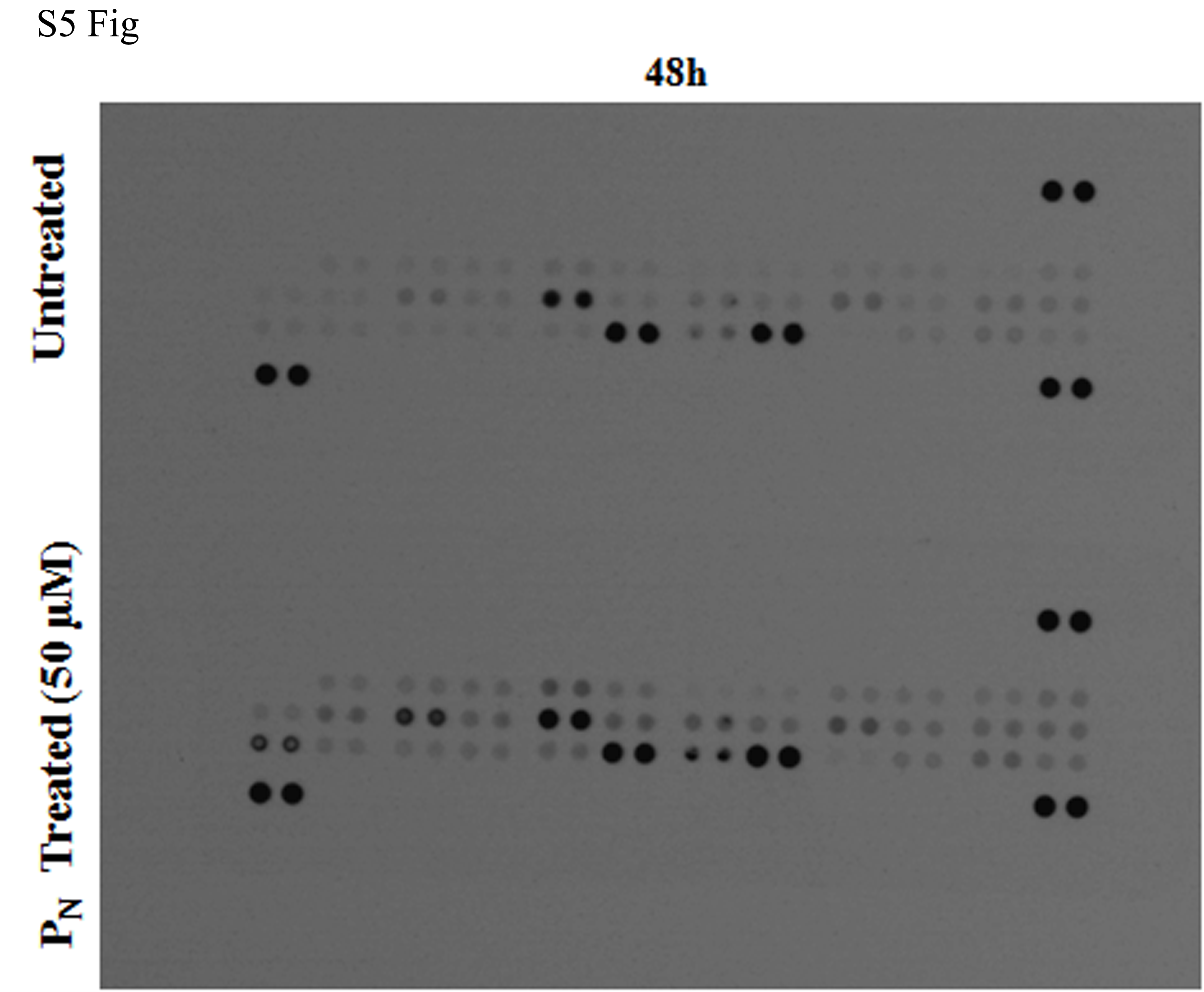

Supplement: S5 Fig — (TIF) [file pone.0191523.s005.tif]
